# Supplementary material for: Clinical Application of Intravitreal Aflibercept Injection for Diabetic Macular Edema Comparing Two Loading Regimens
Source: Medicina (Kaunas). 2023 Mar 12;59(3):558. doi: 10.3390/medicina59030558 (PMC10054468; doi:10.3390/medicina59030558)
Supplement: Supplementary file 1 [file medicina-2187020-supplementary.pdf › medicina-2187020-supplementary.pdf]

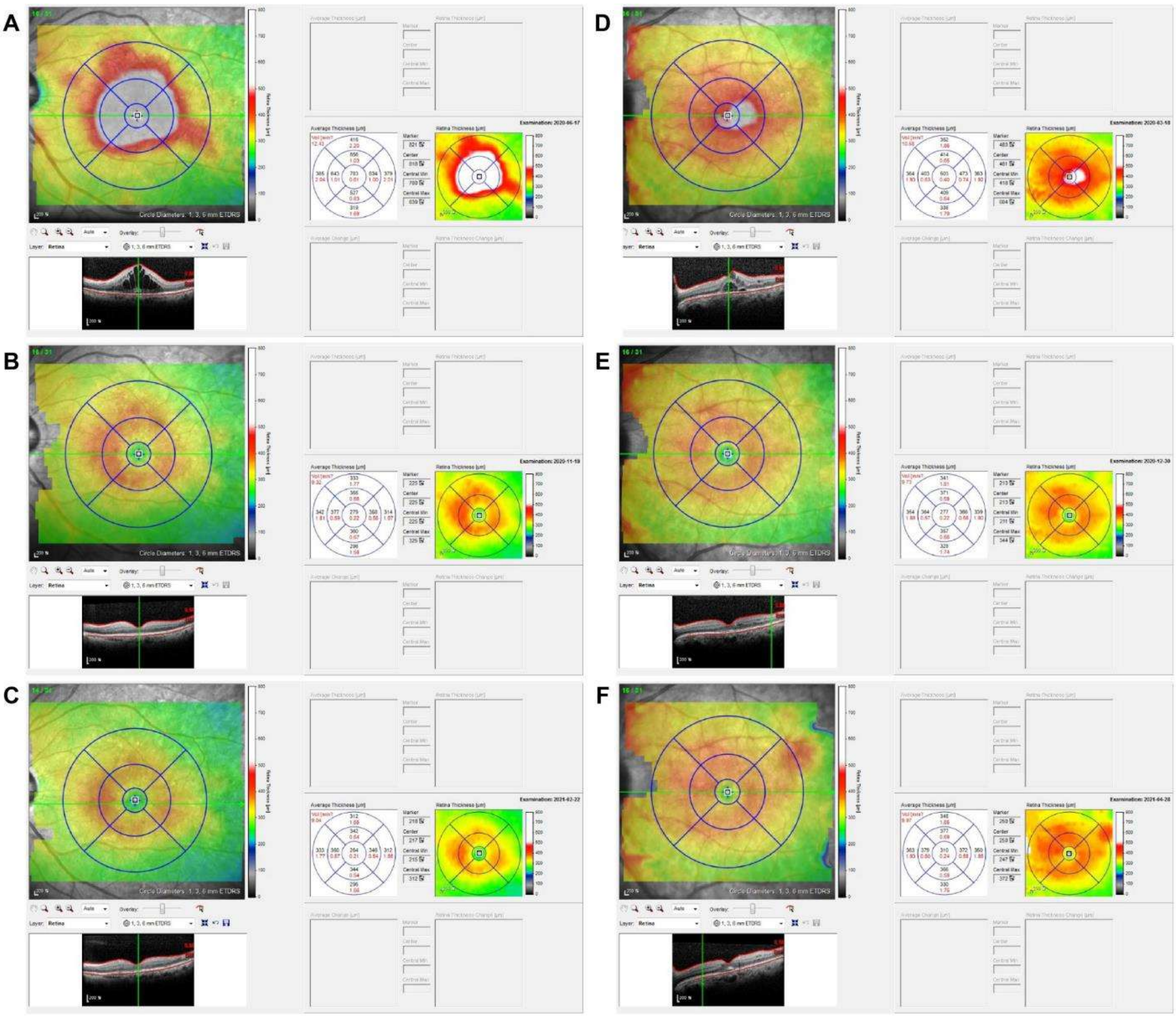

Figure S1. Representative examples of measurements of central retinal thickness (CRT). (**A**) Baseline CRT of a 47-year old male with diabetic macular edema was 783 µm, which was decreased to 279 µm after 3 months (**B**) and to 264 µm after 6 months (**C**) following 3 consecutive monthly loading. (**D**) Baseline CRT of a 49-year old female with diabetic macular edema was 503 µm, which was decreased to 277 µm after 3 months (**E**) and to 310 µm after 6 months (**F**) following 5 consecutive monthly loading.
